# Application of Machine Learning in Developing Decision-Making Support Models for Decompressed Vertebroplasty

**DOI:** 10.3390/healthcare10020214

**Published:** 2022-01-23

**Authors:** Pei-Hung Liao, Yu-Chuan Tsuei, William Chu

**Affiliations:** 1 School of Nursing, National Taipei University of Nursing and Health Sciences, No. 365, Ming-te Road, Peitou District, Taipei 112, Taiwan; peihung@ntunhs.edu.tw; 2 Department of Orthopedics, Cheng Hsin General Hospital, No. 45, Cheng Hsin St., Beitou, Taipei 112, Taiwan; dontspider@yahoo.com.tw; 3 Institute of Biomedical Engineering, National Yang Ming Chiao Tung University, No. 155, Sec. 2, Linong Street, Taipei 112, Taiwan

**Keywords:** decision support, oxygen saturation, risk assessment, roboticists, vertebroplasty

## Abstract

Background: The common treatment methods for vertebral compression fractures with osteoporosis are vertebroplasty and kyphoplasty, and the result of the operation may be related to the value of various measurement data during the operation. Material and Method: This study mainly uses machine learning algorithms, including Bayesian networks, neural networks, and discriminant analysis, to predict the effects of different decompression vertebroplasty methods on preoperative symptoms and changes in vital signs and oxygen saturation in intraoperative measurement data. Result: The neural network shows better analysis results, and the area under the curve is >0.7. In general, important determinants of surgery include numbness and immobility of the lower limbs before surgery. Conclusion: In the future, this association model can be used to assist in decision making regarding surgical methods. The results show that different surgical methods are related to abnormal vital signs and may affect the length of hospital stay.

## 1. Introduction

According to statistics obtained from the World Health Organization, human vertebrae are considered to experience the highest incidence rate of compression fractures caused by osteoporosis, which is more commonly seen in elderly patients, with approximately 27% of patients over 65 years old [1]. Vertebral compression fractures linked to osteoporosis can cause vertebral collapse, spinal deformation, and shrinkage of the abdominal and thoracic space, which could cause a concurrent reduction in pulmonary function and result in restricted mobility and psychological concerns for patients [2,3]. Currently, the commonly used treatment is the use of imaging guidance to inject a cement mixture into the fractured bone (vertebroplasty), while a more modified method includes the insertion of a balloon into the fractured bone to create a space, which is then filled with cement (kyphoplasty) [4,5], and both operations can improve the level of back pain. However, the occurrence of abnormal physiological parameters and decreased oxygen saturation are known to be associated with the selection of different surgical methods, resulting in surgical complications or slower recovery after surgery. In recent years, some doctors have used changed decompressed vertebroplasty, during which they observed that heart rate variability and decreased oxygen saturation occur less frequently [6]. The possible related factors can be used to construct a predictive model through machine learning to facilitate a decision-making support system for future surgical methods.

To improve the quality of life and survival rate of patients, percutaneous vertebroplasty is often used to treat compressive vertebral fractures caused by osteoporosis. However, the most common complication is bone cement leakage. Therefore, many clinical strategies have been proposed to reduce bone cement leakage while maintaining the effect of bone cement in expanding the vertebral body. This study compared traditional and new bone cement delivery technologies and reviewed the clinical effects of the two technologies. This study is the first to apply percutaneous vertebroplasty and hypothesizes that the correlation between preoperative symptoms and intraoperative data changes and surgical methods can be one of the decision-making factors.

## 2. Materials and Methods

### 2.1. Bipedicular Decompressed Kyphoplasty (BiDK)

In the treatment of osteoporotic compression fracture of the vertebral body, cement leakage is a common occurrence in the vertebroplasty procedure, which is due to the porotic bone itself, as well as the fracture line through the cortex. In our new technology, bipedicular decompression percutaneous balloon kyphoplasty (PBK), it was observed that the cement deposition mode followed the laminated pattern that is governed by the rule of the nonlinear Naiver–Stoke equation, which describes the motion of viscous fluid substances. According to the power of the non-Newtonian fluid mechanism, the cement is assumed to be the stress element in the fluid, which is the sum of the diffusing viscous term (proportional to the gradient of velocity) and a pressure term and can be used to describe the viscous flow. As the bone cement mimics the viscosity pattern in the pipe wall regardless of the uniformity of the laminated flow, the bone cement’s viscosity property causes deposits in layers in the vertebral body from the outside to the core body. According to our observation, the bone cement coats the holes caused by the outside crack fracture, which stops the cement from further leaking, and then augments the vertebral body core to complete this procedure.

Traditional bone cement fusion surgery (non-decompression type) may have the complications of high-resolution bi-plane image guide, high-opacity and low-temperature cement, and high-viscosity cement with injection equipment. Thus, the traditional method is relatively passive and conservative. Using negative pressure decompression over the contralateral side can change the bone marrow property and create a target for the cement. When using a balloon to make a larger cavity for cement injection, the negative pressure can be used to control the cement. BiDK, which is an easily controlled material that follows fluid dynamics, can overcome the problem of low-viscosity cement. According to the power of the non-Newtonian fluid mechanism, the cement is assumed to be the stress in the fluid and is the sum of the diffusing viscous term (proportional to the gradient of velocity) and the pressure term. Thus, it can be used to describe the viscous flow. As the bone cement mimics the viscosity pattern in the pipe wall regardless of the uniformity of the laminated flow, the bone cement’s viscosity property causes deposits in layers in the vertebral body from the outside to the core body. In our observation, as the central part of the cement was sucked out by the contralateral negative vacuum decompression, the bone cement coated the holes caused by the outside crack fracture, which then formed a shield to stop the cement from further leaking. Finally, while the bone cement cannot be pulled out by the contralateral portal due to timing, it does augment the vertebral body core to complete this procedure. In general conditions, kyphoplasty using balloon expansion can push the fracture fragments to the peripheral, which shapes a void space, thus, keeping the bone cement in the center of the vertebral body. As we introduced the trocar in the central core of the vertebral body and then expanded the balloon to 3 mL to 4 mL, it hardly caused wall fragments into the canal, which rendered it safer.

### 2.2. Study Design

This study had an exploratory design and used the health information system (HIS) to extract 1136 data of vertebral cases, which were mainly based on the Cross-Industry Standard Process for Data Mining (CRISP) program. Initially, the factors affecting the selection of vertebroplasty and the related postoperative recovery were discussed according to the relevant literature. After comparing the clinical and literature data, the main discriminant factors were selected from the case dataset and integrated into this study as variables to construct a decision-making support system for surgical approaches to vertebroplasty.

This study was conducted according to the guidelines of the Declaration of Helsinki and approved by the research ethics committee of the Cheng Hsin General Hospital (IRB no. (700)108-16). The requirement for informed consent was waived as the data for the study came from the hospital health information database.

### 2.3. Subjects and Data Collection

The primary data source of this study were the medical records of patients who underwent vertebroplasty between 1 January 2011 and 31 December 2018 in the hospitals of northern Taiwan. The subjects of the study were aged between 20 and 80 years old. The data content included age, gender, height, weight, symptoms (soreness, pain, numbness, weakness, and immobility), vital signs, oxygen saturation from the time of bone cement infusion to completion, end-tidal carbon dioxide concentration (ETCO2), and the number of days from surgery to discharge. The collected fields included the original continuity value and the use of normal and abnormal classification fields: (1) systolic blood pressure (SBP) > 140 mm Hg, abnormal; (2) diastolic blood pressure (DBP) < 60 mm Hg, abnormal; (3) arterial oxyhemoglobin saturation (SaO_2_) < 95%, abnormal; (4) pulse rate (PR) > 100/min, abnormal; and (5) respiration rate (RR) ≥ 28/min, abnormal.

### 2.4. Method of Analysis

This study used version 18.2 of the IBM SPSS Modeler software application for data analysis. The selected machine learning methods were conditional Bayesian networks, neural networks, and discriminant analysis. The weights, as determined from the correlational analysis and chi-square testing, were performed on all data. Regarding the items that reached statistically significant differences (*p* < 0.05), their weights were increased for predictive analysis. The contents included age, gender, height, weight, symptoms (soreness, numbness, weakness, and motionlessness), vital signs, blood oxygen saturation of bone cement infusion to completion, and end-tidal carbon dioxide concentration (ETCO2). The normal and abnormal data included (1) systolic blood pressure (SBP) > 140 mm Hg, abnormal; (2) diastolic blood pressure (DBP) < 60 mmHg, abnormal; (3) arterial oxygenated hemoglobin saturation (SaO_2_) < 95%, abnormal; (4) pulse rate (PR) > 100 beats/min, abnormal; and (5) respiratory rate (RR) ≥ 28/min, abnormal.

The Bayesian network, also known as a belief network or the directed acyclic graphical model, is a probabilistic graphical model. The goal of Bayesian classification is to achieve the smallest error through statistical analysis of the probabilities. The use of the probability of known events to infer the category of unknown data is the most significant feature in Bayesian classification [7], and when new sample data are added, a new classification model (probability) can be obtained by adjusting some probabilities; therefore, when data are added continuously, the classification performance improves. However, as the Bayesian classifier is constructed using a probability model, the reasons for the classification (EQ1) in some cases are difficult to explain [7,8]. A Bayesian network can be used to express the probability relationship between a disease and its related symptoms; when a certain symptom is known, a Bayesian network can be used to calculate the probability of various possible diseases [9]. Generally, the nodes in the Bayesian network represent random variables, which can be observable variables, latent variables, or unknown parameters. The arrow connecting any two nodes indicates that the two variables have a causal or independent relationship, while two nodes connected by a single arrow imply that one of the nodes is a “parent” and the other is a “descendant or child” and these two nodes will produce a conditional probability value [7].

Posterior Probability (P(H/X))
Prior Probability (P(H))

where X is data tuple and H is some hypothesis.

P(H/X) = P(X/H)P(H)/P(X)(1)

A neural network is also an adaptive system for estimating functions. Learning capability is a nonlinear statistical data-modeling tool and is usually optimized by a learning method based on mathematical statistics [8]. A neural network is a machine learning method that imitates the operation of the brain and is mainly composed of neurons and synapses, where neurons are on different layers. The neurons between different layers have bonds of different strengths. Neural networks imitate this structure and are developed based on this concept [9]. In the field of the perception of artificial intelligence, neural networks are adopted for identification, decision-making, control, prediction, and other tasks. The layers of a neural network usually include an input layer, a hidden layer, and an output layer, and more than one hidden layer can exist. In particular, a neural network with two or more hidden layers is generally referred to as a deep neural network [9,10].

Discriminant analysis is a method used in multivariate statistical analysis to discriminate the type (population) of the sample (respondents) [7,11]. It classifies similar samples (respondents) into one category (population); derives one or a group of differentiable (discriminant) functions based on the sample data; and specifies a discriminant rule to determine the category of the sample to be discriminated, in order to minimize the error rate [10,12].

## 3. Results

### 3.1. Demographic Data Analysis

As shown in Table 1, the average age of the 1136 cases adopted in this study was 55.89 years old, with more female case files (65.6%) than those of males. The symptoms were mostly pain (91.7%) and weakness (66.5%). Regarding etiological analysis, the most common active posture was half standing-up and half sitting-down. Regarding the severity of pain, as measured by the standard pain scale, most patients scored an average of 6.5 (moderate pain). The average values of vital signs and oxygen saturation were as follows: a body temperature of 36.308, a pulse rate of 81.58, a respiration rate of 21.31, systolic blood pressure of 149.37, and a diastolic blood pressure of 78.26. The blood oxygen saturation was 92–100% in the first minute of bone cement injection, 97–100% in the tenth minute, and 89–100% when the cement hardened. Intraoperative bone cement leakage was 7% in vertebroplasty and less than 0.07% in kyphoplasty.

### 3.2. Association Model of Vertebroplasty and Various Factors

#### 3.2.1. Bayesian Network

**Figure 1:**
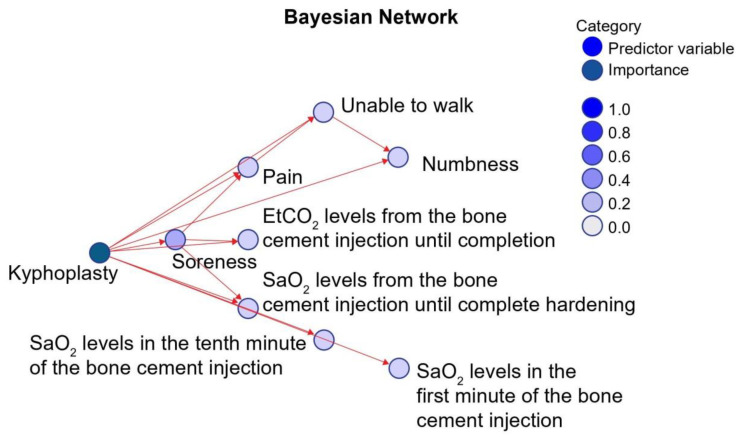
Bayesian network model of the impact factors of modified vertebroplasty (kyphoplasty).

Through the conditional probability analysis of the 17 factors using the Bayesian network, the overall probability of choosing vertebroplasty was 80%. Analysis revealed the following impact factors as crucial: physiological symptoms, such as soreness, numbness, immobility, and SaO_2_ in the first minute of bone cement injection. Other minor factors were pain and ETCO2 after bone cement injection (Figure 1).

#### 3.2.2. Neural Network

**Figure 2:**
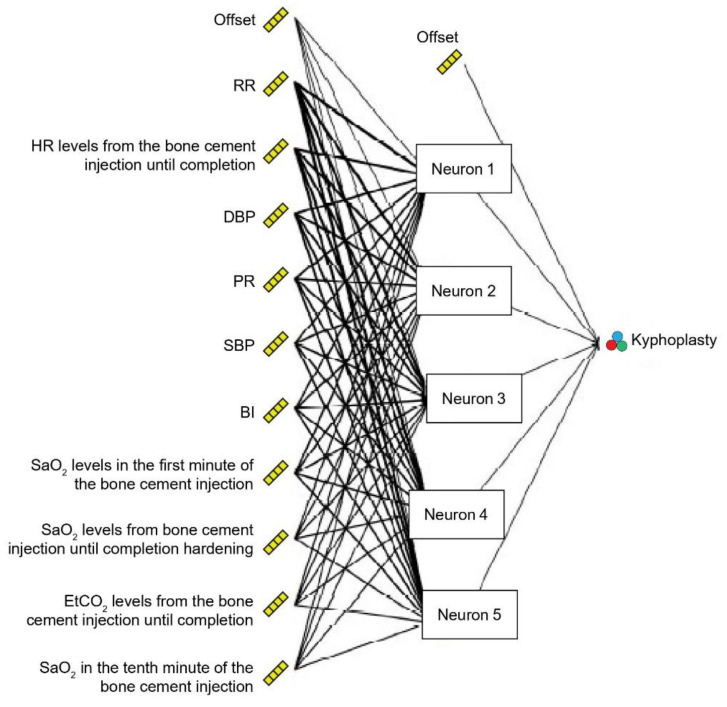
Neural network model of the impact factors of modified vertebroplasty (kyphoplasty).

**Figure 3:**
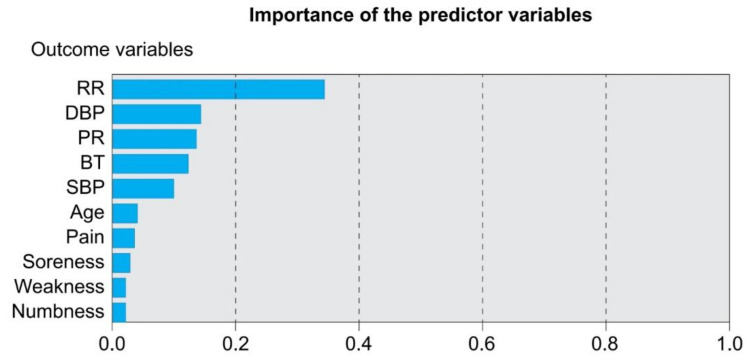
Important impact factors suggesting modified vertebroplasty (kyphoplasty).

This study focused on a general prediction model. Thus, only one layer was set as the hidden layer of the artificial neural network. The original data of the 17 factors (gender, age, pain score, symptoms, characteristics, etc.), as shown in Table 1, were input into the input layer, while signal neurons were set in the hidden layer for testing. The output layer contained the choice of surgery, meaning the conventional or the modified vertebroplasty procedure. The learning rate was set between 0.15 and 0.25; a relatively low learning rate usually results in better learning results, and a convergent result cannot be obtained using a learning rate greater than 0.25. The termination condition of network training uses the root-mean-square error (RMSE) to measure the deviation between the observed and true values; therefore, the minimum RMSE of the test data was chosen to be ≤0.00001 to form the final network structure. The results show that the overall accuracy was 83.5%. The most important factors influencing the choice of different methods of vertebroplasty are the number of breaths, changes in heartbeat caused by bone cement infusion, and diastolic blood pressure, as shown in Figure 2 and Figure 3.

#### 3.2.3. Discriminant Analysis

This study used surgical method classification as the representative of two clusters. Then, the original 17 variables were used to conduct discriminant analysis to establish a classification table (Table 2), which showed 76.4% as the correct discrimination rate of the clustering results and implies highly consistent internal homogeneity for each cluster. A comparison of the differences between the use of kyphoplasty and modified vertebroplasty shows a strong correlation of kyphoplasty with numbness, immobility, and SaO_2_ in the first minute of bone cement injection, meaning a significant difference. Vertebroplasty had a higher correlation with pain, immobility, low DBP abnormalities, and excessive HR. As shown in Table 1, kyphoplasty has higher discriminant accuracy than vertebroplasty and the difference is statistically significant (t = −4.62; *p* < 0.001).

### 3.3. Comparison of the Areas under the ROC Curves of the Three Models

**Figure 4:**
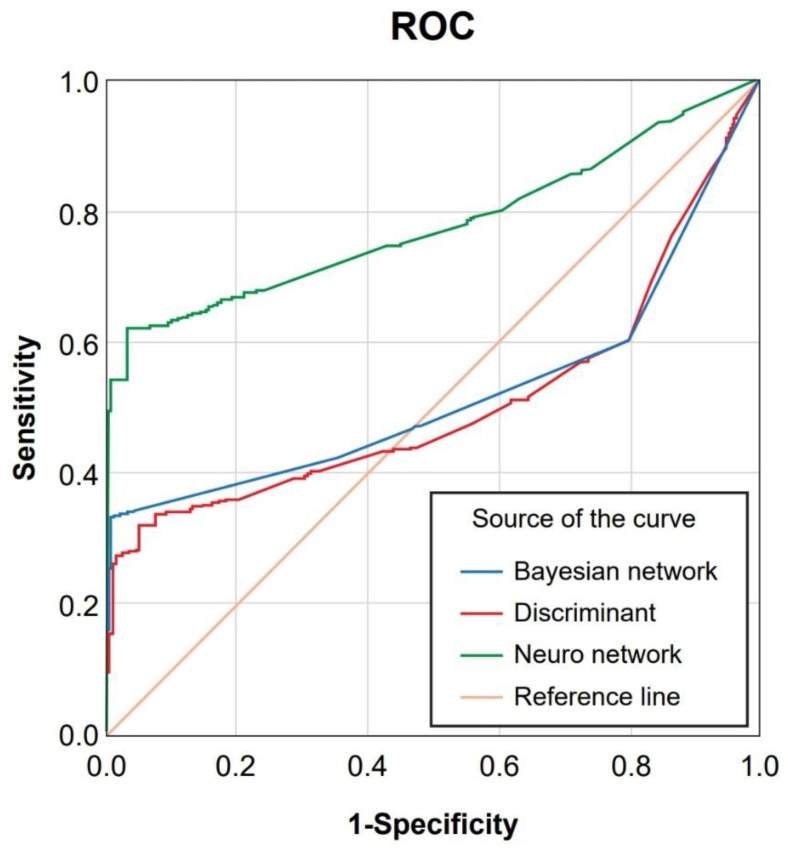
Areas under the receiver operating characteristic (ROC) curves of the three models.

Table 3 and Figure 4 show the receiver operating characteristic (ROC) curves of the three models. As shown, the area under the ROC curve (AUC) of the neural network is better, reaching 0.77. When the AUC > 0.5, the classification effect of the classifier is better than that of stochastic guessing and the model achieves a predictive value.

## 4. Discussion

In the past, the choice of surgical technique was determined by an individual’s age, preoperative symptoms, and the physician’s personal habits [13,14,15]. This study investigated the choice of surgery based on personal symptoms and physiological parameters, as well as intraoperative blood oxygen saturation and postoperative recovery. The analysis results of the three models show that some factors, such as pain level and age, are not among the important impact factors, which may be due to the previous neglect of vital sign changes during surgical methods and the influence of blood oxygen saturation on the postoperative period. The incidence rate of vertebroplasty was 7% higher than the probability of intraoperative bone cement leakage. Bone cement leakage is considered to be positively correlated with blood oxygen saturation and might be correlated with postoperative recovery and the duration of hospitalization [16].

**Figure 5:**
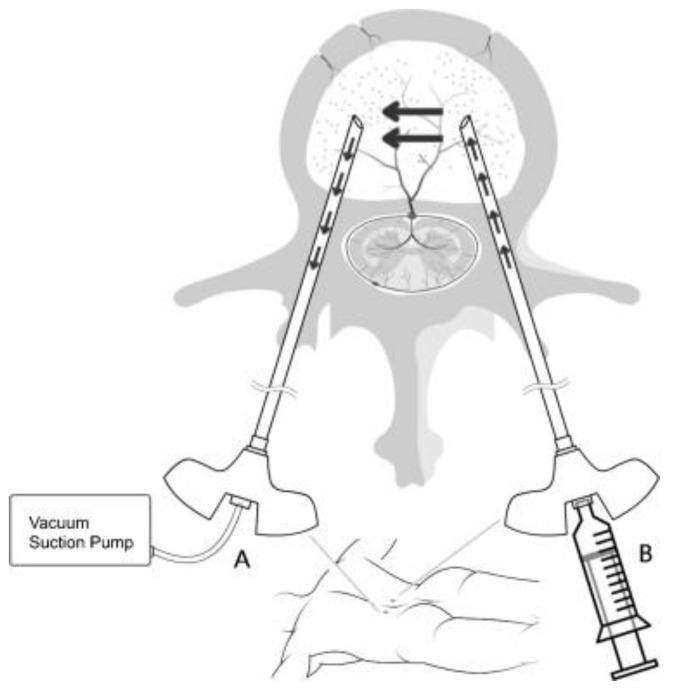
Bipedicular decompressed PBK.

According to the power of the non-Newtonian fluid mechanism, the cement is assumed to be the stress in the fluid and is the sum of the diffusing viscous term (proportional to the gradient of velocity) and a pressure term, which can be used to describe the viscous flow. As the bone cement mimics the viscosity pattern in the pipe wall regardless of the uniformity of the laminated flow, the bone cement’s viscosity property causes deposits in layers in the vertebral body from the outside to the core body. According to our observation, when the central part of the cement is sucked out by contralateral negative vacuum decompression, the bone cement coats the holes caused by the outside crack fracture, which forms a shield to stop the cement from further leakage. Finally, while the bone cement cannot be pulled out by the contralateral portal due to timing, it would augment the vertebral body core to complete this procedure. As this surgical method is a relatively new method, there is little literature tracking the decisions and results of different surgical methods. During bipedicular decompressed PBK, the acrylic bone cement is injected with a simultaneous application of continuous negative suction pressure on the contralateral side and this negative pressure creates a pulling force that attracts the bone cement by laminating the flow pattern deposition within the vertebral body [17,18] (Figure 5).

The three prediction models performed well in resolving the two types of vertebroplasty procedures (the AUC ranged from 0.51 to 0.78). As the sensitivity, specificity, and accuracy of the three models were all above 70%, the records of missing or unknown data during the modeling process were excluded and the accuracy of the neural network model was concluded to outperform discriminant analysis and the Bayesian network. While other methods, such as replacing average and model values, can be used to prepare datasets for modeling, due to the possible uncertainty of such alternative methods, a conservative method to exclude the data was selected, and this result can be verified in future follow-up research to facilitate the elimination of possible bias.

In addition to its advantages in result prediction, it is also easier to understand the interpretation method of the neural network model. According to the magnitude of the coefficients of the comparison function, the weight of each variable and its degree of contribution to the model are known [19,20]. As a judgment based on conditional rules provided by the Bayesian network is rather vague and the method of interpretative prediction provided by discriminant analysis is not effective enough, it is worth considering the use of an integrated method for predictive models, such as the combination of discriminant analysis and neural networks, which will further improve the accuracy and interpretation capabilities of the model [21,22]. Other machine learning methods, such as deep learning or XGBoost, should also be considered as possible options in the future. Further development of mobile application programs or models packaged as a web service can be considered, and the model can be modified by dynamically updating the dataset in the database, which can be provided to physicians as a model that assists them in their optimal decision making [23,24,25].

At present, decision making regarding difficult decisions often depends on the shared medical experiences of physicians, and treatment options should also be guided by empirical evidence to provide the best solution for each case. In our follow-up study, we hope to identify a physician and an actual case to apply the findings of this study and choose the most suitable surgical method based on the symptoms and personal conditions of the case.

Limitation: Due to the use of data analysis, it was impossible to learn the data regarding the qualitative feelings of the cases. As feeling-related data may increase the accuracy of the prediction model, future studies may be expanded to include qualitative data.

## 5. Conclusions

This study was aimed at exploring the optimal decision-making assistance model for surgical methods and analyzing the performance of three models: Bayesian network, neural network, and discriminant analysis. While the findings show that all three models have a certain degree of accuracy in predicting the choice of lumbar decompression surgery, the accuracy of the neural network model was observed to outperform those of discriminant analysis and the Bayesian network. Therefore, as the neural network model has good predictive analysis and classification capability and provides major insights into the weights of the variables, it is the most preferred method for the dataset of this study. The advantage of the classification model is that it can be used as a low-cost preliminary diagnosis reference. However, for the development of classification models and clinical practices, there are still high inspection costs due to over-diagnosis or the accurate identification of acute and chronic conditions, which are problems that need to be solve. Considering the complexity and significance of medical applications, our future study will continue to develop models that can use the data and algorithms more comprehensively.

**Figure 6:**
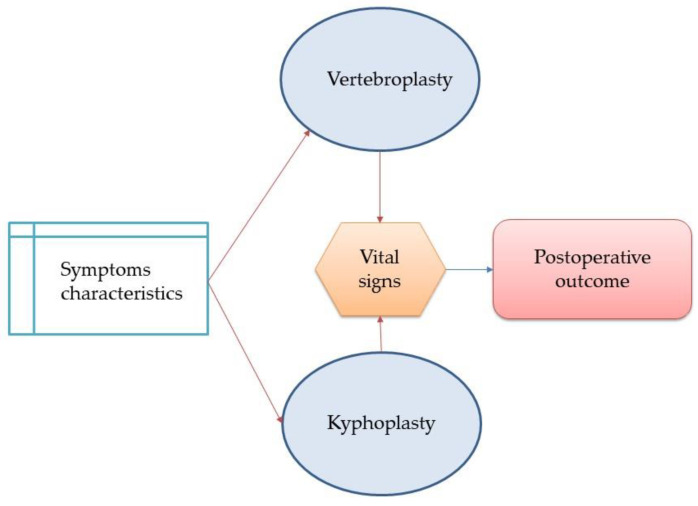
Surgical methods and the preoperative and intraoperative association model.

Moreover, this analysis method allows for similar and popular results to be included according to the shared experiences of physicians. This model can provide effective guidance for identifying surgical methods with similar symptoms and possible complications, assist physicians in the surgical selection, and reduce subjective cognition and judgment. In addition, it can reduce unnecessary risks and shorten the length of hospital stay, which will help improve the quality of medical care and reduce the waste of medical resources. In the future, further clinical tests can be conducted, more data can be collected to verify the model accuracy, and the efficiency of other deep learning methods can be compared in order to provide the most suitable clinical care model. This study developed a decision support machine learning model according to the parameters considered during preoperative and postoperative surgical procedures. As shown in Figure 6, the characteristics and effects of preoperative pain are the first step in the decision making of surgical methods and the surgical method, intraoperative breathing, and blood pressure changes are correlated with the results of postoperative recovery.

## Figures and Tables

**Table 1:** Demographic data analysis (*n* = 1136).

Demographic Characteristic	Mean (SD)	*n* (%)	95% Confidence Interval/*p*-Value
Gender			/0.478
Male		390 (34.4)	
Female		746 (65.6)	
Age (years old)	56.89 (12.6)		29–78
Pain score	6.5 (1.2)		3–9
Common posture			/0.067
Stand		585 (51.5)	
Sit		551 (48.5)	
Symptom characteristics			/0.002 **
Pain		1043 (91.7)	
Soreness		626 (55.1)	
Numbness		700 (71.6)	
Unable walk		623 (51.8)	
Powerless		756 (66.5)	
Vital signs			/0.023 **
Temperature	36.308 (16.69)		35.4–37.2
Pulse rate	81.58 (16.74)		60–103
Respiration rate	21.31 (1.007)		13–25
Systolic blood pressure	149.47 (31.52)		115–202
Diastolic blood pressure	78.26 (17.34)		55–106
SaO_2_ in the 1st minute of bone cement injection	99.29 (1.7)		92–100
SaO_2_ in the 10th minute of bone cement injection	99.94 (0.26)		97–100
Bone cement injection until SaO_2_ hardening	98.24 (5.8)		89–100
Intraoperative bone cement leakage			/0.047 **
Vertebroplasty		37 (7% = 37/497) *	
Kyphoplasty		5 (0.07% = 5/639) *	
Length of postoperative hospital stay			/0.003 **
Vertebroplasty	3.2 (0.6)		
Kyphoplasty	2.1 (1.1)		

* (Number of cases/Total number); ** *p* < 0.05.

**Table 2:** Discriminant analysis for operation classification (*n* = 1136).

Classification	Kyphoplasty %	Vertebroplasty %	Number
Kyphoplasty	506	133	639
	79%	21%	
Vertebroplasty	135	362	497
	27.2%	72.8%	
Total	641	495	1136

Correct classification rate: (506 + 362)/1136 = 76.4%.

**Table 3:** Areas under the curves (AUC) of the three models.

Verification Result Variable	Area	Standard Error	Significance	95% CI
Bayesian network	0.52	0.019	0.223	0.483~0.559
Neural network	0.78	0.015	0.000	0.748~0.808
Discriminant analysis	0.51	0.019	0.674	0.470~0.544

## Data Availability

The data presented in this study are available on request from the corresponding author. The data are not publicly available due to IRB restrictions. Data can be made available upon reasonable request.

## References

[1] Feng L., Feng C., Chen J., Wu Y., Shen J.M. (2018). The risk factors of vertebral refracture after kyphoplasty in patients with osteoporotic vertebral compression fractures: A study protocol for a prospective cohort study. BMC Musculoskelet. Disord..

[2] Buchbinder R., Johnston R.V., Rischin K.J., Homik J., Jones C.A., Golmohammadi K., Kallmes D.F. (2018). Percutaneous vertebroplasty for osteoporotic vertebral compression fracture. Cochrane Database Syst. Rev..

[3] Chu W., Tsuei Y.C., Liao P.H., Lin J.H., Chou W.H., Chu W.C., Young S.T. (2013). Decompressed percutaneous vertebroplasty: A secured bone cement delivery procedure for vertebral augmentation in osteoporotic compression fractures. Injury.

[4] Gambacciani M., Levancini M. (2014). Management of postmenopausal osteoporosis and the prevention of fractures. Panminerva Med..

[5] Ballane G., Cauley J.A., Luckey M.M., El-Hajj Fuleihan G. (2017). Worldwide prevalence and incidence of osteoporotic vertebral fractures. Osteoporos. Int..

[6] Byun J.H., Jang S., Lee S., Park S., Yoon H.K., Yoon B.H., Ha Y.C. (2017). The efficacy of bisphosphonates for prevention of osteoporotic fracture: An update meta-analysis. J. Bone Metabol..

[7] Liao S.H., Wen C.H. (2019). Data Mining: Artificial Intelligence and Machine Learning Development.

[8] Chien P.L., Liu C.F., Huang H.T., Jou H.J., Chen S.M., Young T.G., Wang Y.F., Liao P.H. (2021). Application of artificial intelligence in the establishment of an association model between metabolic syndrome, TCM constitution, and the guidance of medicated diet care. Evid.-Based Complement. Alternat. Med..

[9] Ahuja A.S. (2019). The impact of artificial intelligence in medicine on the future role of the physician. Peer J..

[10] Liao P.H., Hsu P.T., Chu W., Chu W.C. (2015). Applying artificial intelligence technology to support decision-making in nursing: A case study in Taiwan. Health Inform. J..

[11] Halilaj E., Rajagopal A., Fiterau M., Hicks J.L., Hastie T.J., Delp S.L. (2018). Machine learning in human movement biomechanics: Best practices, common pitfalls, and new opportunities. J. Biomech..

[12] Phinyomark A., Petri G., Báñez-Marcelo E., Osis S.T., Ferber R. (2018). Analysis of big data in gait biomechanics: Current trends and future directions. J. Med. Biol. Eng..

[13] Chen Y.C., Zhang L., Li E.N., Ding L.X., Zhang G.A., Hou Y., Yuan W. (2019). Unilateral versus bilateral percutaneous vertebroplasty for osteoporotic vertebral compression fractures in elderly patients: A meta-analysis. Medicine.

[14] Balkarli H. (2015). Treatment of osteoporotic vertebral compression fractures with percutaneous vertebroplasty under local anesthesia: Cinical and radiological results. Int. J. Clin. Exp. Med..

[15] Fisher C., Tee J. (2016). Editorial: The utility of the modified frailty index for risk stratification in patients undergoing spine surgery. J. Neurosurg. Spine.

[16] Mei J., Wu D., Song X.X., Liu Q. (2020). Comparison of early results of vesselplasty and percutaneous vertebroplasty in the treatment of elderly patients with osteoporotic vertebral compression fracture. Res. Sq..

[17] Nikoobakht M., Gerszten P.C., Shojaei S.F., Shojaei H. (2021). Percutaneous balloon kyphoplasty in the treatment of vertebral compression fractures: A single-center analysis of pain and quality of life outcomes. Br. J. Neurosurg..

[18] Francio T.V., Gill B., Rupp A., Sack A., Sayed D. (2021). Interventional procedures for vertebral diseases: Spinal tumor ablation, vertebral augmentation, and basivertebral nerve ablation—A scoping review. Healthcare.

[19] MacIntyre N.J., Thabane L., Papaioannou A., Giangregorio L.M., Skidmore C.J. (2019). Exercise for improving outcomes after osteoporotic vertebral fracture. Cochrane Database Syst. Rev..

[20] (2017). Understanding the Basics of Clinical Decision Support Systems. https://healthitanalytics.com/features/understanding-the-basics-of-clinical-decision-support-systems#.

[21] Choi K.S. (2020). Integrating artificial intelligence into healthcare research. J. Nurs..

[22] Negm A.E., Kandil A.H., Hassan O. (2017). Decision support system for lymphoma classification. Curr. Med. Imaging Rev..

[23] Aruna D.B., Rajaekaran M.P. (2020). Medical decision support system for brain image classification. Eur. J. Mol. Clin. Med..

[24] Kang J., Ullah Z., Gwak J. (2021). MRi-based brain tumor classification using ensemble of deep features and machine learning classifiers. Sensors.

[25] Chu W., Ho C.S., Liao P.H. (2021). Comparison of different predicting models to assist the diagnosis of spinal lesions. Inform. Health Soc. Care.

